# Parkin Is Protective against Proteotoxic Stress in a Transgenic Zebrafish Model

**DOI:** 10.1371/journal.pone.0011783

**Published:** 2010-07-30

**Authors:** Mareike E. Fett, Anna Pilsl, Dominik Paquet, Frauke van Bebber, Christian Haass, Jörg Tatzelt, Bettina Schmid, Konstanze F. Winklhofer

**Affiliations:** 1 Neurobiochemistry, Adolf-Butenandt-Institute, Ludwig Maximilians University, Munich, Germany; 2 German Center for Neurodegenerative Diseases (DZNE), Munich, Germany; 3 Biochemistry, Adolf-Butenandt-Institute, Ludwig Maximilians University, Munich, Germany; National Institutes of Health, United States of America

## Abstract

**Background:**

Mutations in the gene encoding the E3 ubiquitin ligase parkin (PARK2) are responsible for the majority of autosomal recessive parkinsonism. Similarly to other knockout mouse models of PD-associated genes, parkin knockout mice do not show a substantial neuropathological or behavioral phenotype, while loss of parkin in *Drosophila melanogaster* leads to a severe phenotype, including reduced lifespan, apoptotic flight muscle degeneration and male sterility. In order to study the function of parkin in more detail and to address possible differences in its role in different species, we chose *Danio rerio* as a different vertebrate model system.

**Methodology/Principal Findings:**

We first cloned zebrafish parkin to compare its biochemical and functional aspects with that of human parkin. By using an antisense knockdown strategy we generated a zebrafish model of parkin deficiency (knockdown efficiency between 50% and 60%) and found that the transient knockdown of parkin does not cause morphological or behavioral alterations. Specifically, we did not observe a loss of dopaminergic neurons in parkin-deficient zebrafish. In addition, we established transgenic zebrafish lines stably expressing parkin by using a Gal4/UAS-based bidirectional expression system. While parkin-deficient zebrafish are more vulnerable to proteotoxicity, increased parkin expression protected transgenic zebrafish from cell death induced by proteotoxic stress.

**Conclusions/Significance:**

Similarly to human parkin, zebrafish parkin is a stress-responsive protein which protects cells from stress-induced cell death. Our transgenic zebrafish model is a novel tool to characterize the protective capacity of parkin *in vivo*.

## Introduction

Parkinson's Disease (PD), the second most common neurodegenerative disease after Alzheimer's Disease, is characterized primarily by the progressive loss of dopaminergic neurons in the substantia nigra and subsequent dopamine depletion in the striatum. The etiopathogenesis of sporadic PD is only poorly understood, thus a milestone in PD research has been the identification of monogenic familial variants which account for up to 10% of PD cases. Mutations in the genes encoding α-synuclein and LRRK2 (leucine-rich repeat kinase 2) are responsible for autosomal dominant forms of PD, presumably by a gain-of-function mechanism. Loss-of-function mutations in the genes encoding parkin, PINK1, and DJ-1 mediate autosomal recessive PD. Sporadic and monogenic forms share important clinical, pathological and biochemical features, notably the progressive demise of dopaminergic neurons in the substantia nigra. Therefore, insight into the function and dysfunction of PD-associated gene products can help to elucidate the underlying mechanisms leading to neuronal cell death.

A large number and a wide spectrum of mutations in the parkin gene has been identified in PD patients, which account for the majority of early onset, autosomal recessive parkinsonism. The parkin gene encodes a cytosolic protein of 465 amino acids with a ubiquitin-like domain (UBL) at the N-terminus and a RBR (RING between RING) domain close to the C-terminus, consisting of two RING finger motifs which flank a cysteine-rich in-between RING finger domain. The presence of the RBR domain suggested that parkin has an E3 ubiquitin ligase activity, mediating the covalent attachment of ubiquitin to substrate proteins. A well known function of E3 ubiquitin ligases is to target substrate proteins for degradation by the proteasome. However, there is increasing experimental evidence that parkin can induce proteasome-independent ubiquitylation and that this mode of ubiquitylation may underlie the wide neuroprotective activity of parkin [1], [2], [3], [4], [5] To promote the understanding of the physiological function of parkin, great efforts have been made to establish animal models. Loss of parkin expression in mice accomplished by the deletion of exon 2, 3 or 7 did not recapitulate the typical characteristics of parkinsonism. Notably, only mild alterations in dopaminergic neurotransmission have been observed, but no degeneration of dopaminergic neurons, which might be explained by functional redundancy or compensatory mechanisms [6], [7], [8], [9]. In contrast to parkin knockout mice, *Drosophila* parkin null mutants show a marked phenotype, such as reduced lifespan, male infertility and locomotor defects caused by apoptotic muscle degeneration [10], [11], [12]. This discrepancy prompted us to investigate the role of parkin in another model organism. The zebrafish (*Danio rerio*) is emerging as a convenient vertebrate model to unravel the function of genes and to establish models for human diseases [13]. Considerable advantages are the external development of embryos and optical clarity during embryogenesis which facilitate the analysis of early developmental processes as well as disease progression. Furthermore, genetic manipulation, like a transient gene knockdown, can easily be performed by microinjection of antisense oligonucleotides at the single-cell-stage. To determine the impact of environmental influences, chemical compounds or toxins can be administered to the surrounding media.

In this study we analyzed the zebrafish orthologue of the human parkin gene using *in vitro* and *in vivo* approaches. We show that human and zebrafish parkin share key biochemical and functional features. Similarly to human parkin, zebrafish parkin shows auto-/transubiquitylation activity and is prone to misfolding and aggregation under high level stress conditions. Moreover, both human and zebrafish parkin are transcriptionally up-regulated in response to mild stress and can protect cells from stress-induced cell death. A transient knockdown of parkin in zebrafish revealed that parkin is not essential for its development. However, parkin deficiency increases the vulnerability of zebrafish to proteotoxic stress, while transgenic zebrafish stably overexpressing parkin are less sensitive to cell death induced by proteotoxicity.

## Results

### The parkin orthologue is highly conserved in zebrafish and expressed throughout development

**Figure 1:**
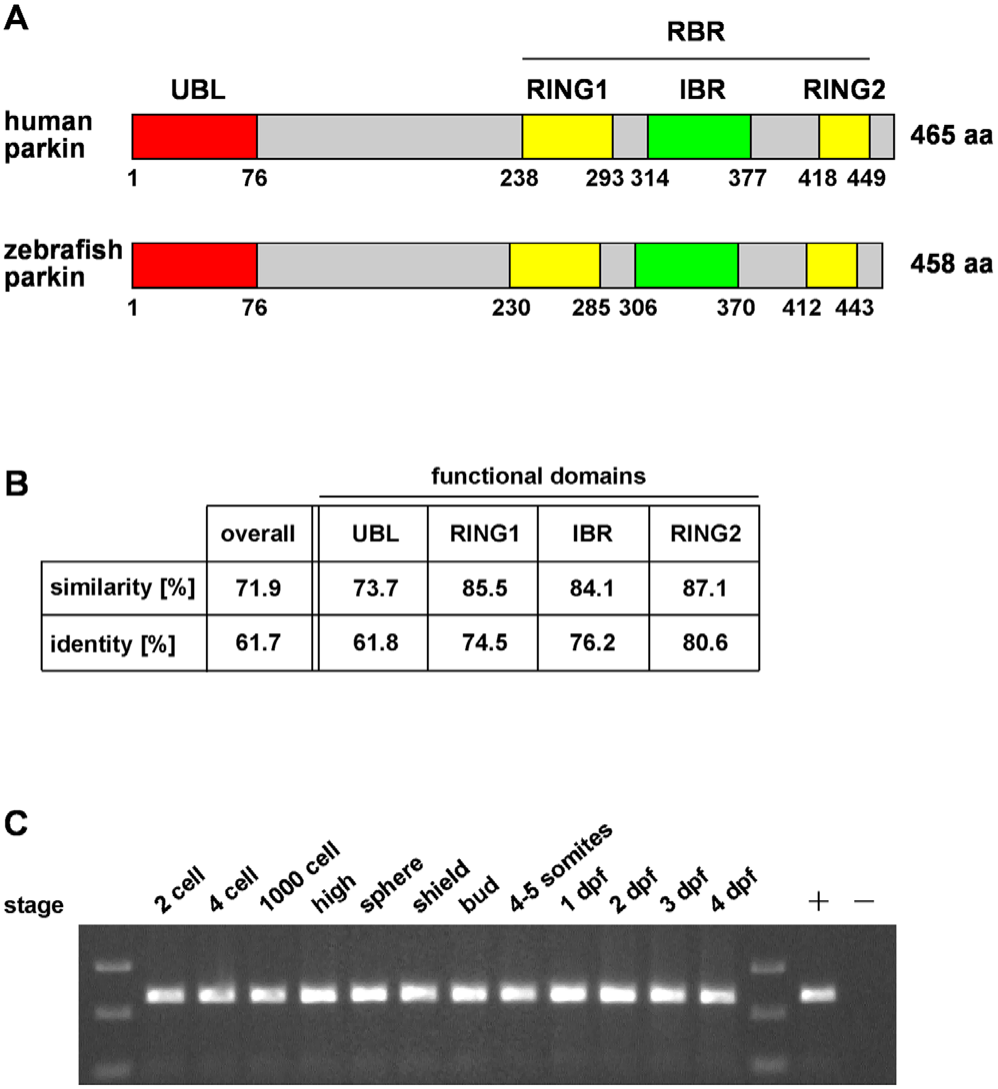
The zebrafish parkin orthologue is highly conserved and expressed during development. (**A**) Modular structure of human and zebrafish parkin. UBL: ubiquitin-like domain, RING: really interesting new gene, IBR: in-between RING, RBR: RING between RING fingers. (**B**) Protein sequence alignment of zebrafish parkin and human parkin was analyzed in regard to similarity and identity using BOXSHADE 3.21. Analysis of the functional domains of parkin reveals a higher degree of identity/similarity compared to the overall sequence. (**C**) Parkin mRNA expression in developing zebrafish. Total mRNA was reversely transcribed into cDNA and analyzed by PCR using parkin-specific primers; + positive control: zebrafish parkin plasmid DNA as a template; - negative control without template. dpf: days post fertilization.

BLAST search in the ENSEMBL zebrafish database using the human parkin protein sequence (NP_054642) led to the identification of one parkin orthologue in zebrafish (ENSDARG00000021555, zgc: 112390). The zebrafish parkin cDNA comprises an open reading frame of 1377 base pairs, encoding a 458 amino acid protein (Fig. 1A). The parkin protein is highly conserved between zebrafish and man; the overall identity is 62% with a significant increase in identity (up to 87%) in the functional domains (Fig. 1B). Reverse transcription PCR was used to analyze the expression profile of parkin. We detected parkin-specific transcripts during all stages of zebrafish development (Fig. 1C).

### Zebrafish parkin and human parkin share key biochemical features

A clone containing the zebrafish parkin cDNA was obtained from the Deutsches Ressourcenzentrum für Genomforschung (RZPD) and verified by sequencing. Despite the high level of identity, no anti-parkin antibody is available so far that crossreacts with zebrafish parkin. To circumvent this problem, the coding sequence of zebrafish parkin was subcloned into the pCMV Tag 2B vector (Stratagene), which allows the expression of N-terminally FLAG-tagged zebrafish parkin and subsequent detection with an anti-FLAG-antibody.

**Figure 2:**
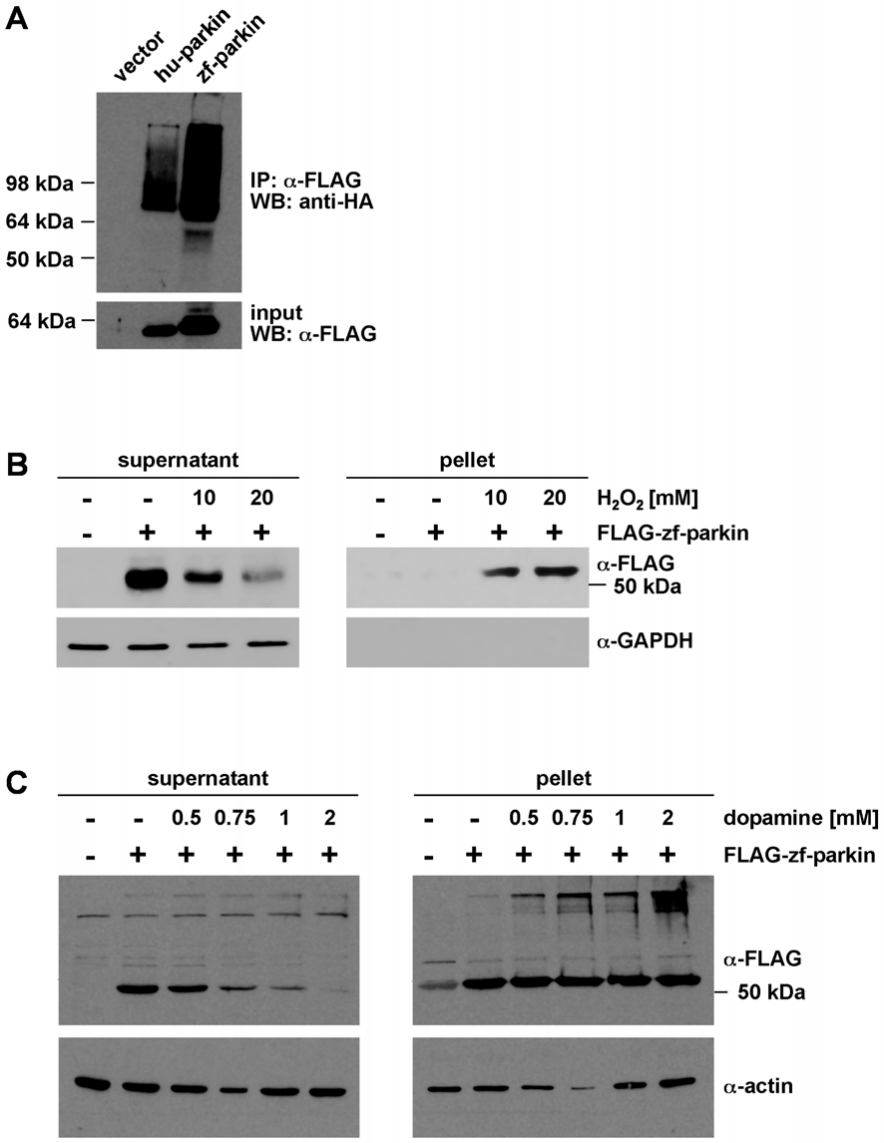
Zebrafish parkin promotes auto-/transubiquitylation and is prone to misfolding in the presence of high level oxidative stress. (**A**) Zebrafish parkin shows auto-/transubiquitylation activity. HEK293T cells were co-transfected with HA-ubiquitin and either FLAG-tagged human parkin (hu-parkin) or FLAG-tagged zebrafish parkin (zf-parkin). The cells were treated with the proteasomal inhibitor MG-132 overnight. At 24 h after transfection, cells were harvested, lysed under denaturing conditions, and cleared by centrifugation. The supernatants were subjected to an immunoprecipitation (IP) using anti-FLAG agarose beads. The immunoprecipitates were resolved by SDS-PAGE and immunoblotted with an anti-HA mAB (WB). Aliquots of the supernatants were removed before immunoprecipitation and were immunoblotted with anti-FLAG mAB (input, lower panel). (**B**) SH-SY5Y cells were transiently transfected with FLAG-tagged zebrafish parkin (FLAG-zf-parkin) and exposed to oxidative stress (10 and 20 mM H_2_O_2_, 30 min). The cells were then lysed in detergent buffer (0.1% Triton X-100 in PBS), and fractionated into detergent-soluble (supernatant) and -insoluble (pellet) fractions by centrifugation. Parkin present in the supernatant and pellet fraction was analyzed by Western blotting using an anti-FLAG monoclonal antibody. GAPDH (only present in the soluble fraction) was used as a loading control. (**C**) SH-SY5Y cells were transiently transfected with FLAG-tagged zebrafish parkin (FLAG-zf-parkin) and exposed to dopamine stress (0.5, 0.75, 1 and 2 mM, 5 h). The cells were then lysed in detergent buffer (0.1% Triton X-100 in PBS), and detergent-soluble and -insoluble fractions were analyzed as described in (B). Actin was used as a loading control.

We first analyzed zebrafish parkin in cultured cells and focussed on biochemical and functional aspects of parkin. First, we tested whether zebrafish parkin has an E3 ubiquitin ligase activity. Since the discussion on authentic parkin substrates is still ongoing and controversial, we made use of the auto-/transubiquitylation activity observed for human parkin. HEK293T cells were co-transfected with HA-tagged ubiquitin and either FLAG-tagged human or zebrafish parkin. Immunoprecipitation under denaturing conditions was performed with anti-FLAG agarose beads, and precipitated proteins were subjected to a Western blot analysis using an anti-HA antibody. Similarly to human parkin, zebrafish parkin was able to promote its auto-/transubiquitylation (Fig. 2A).

**Figure 3:**
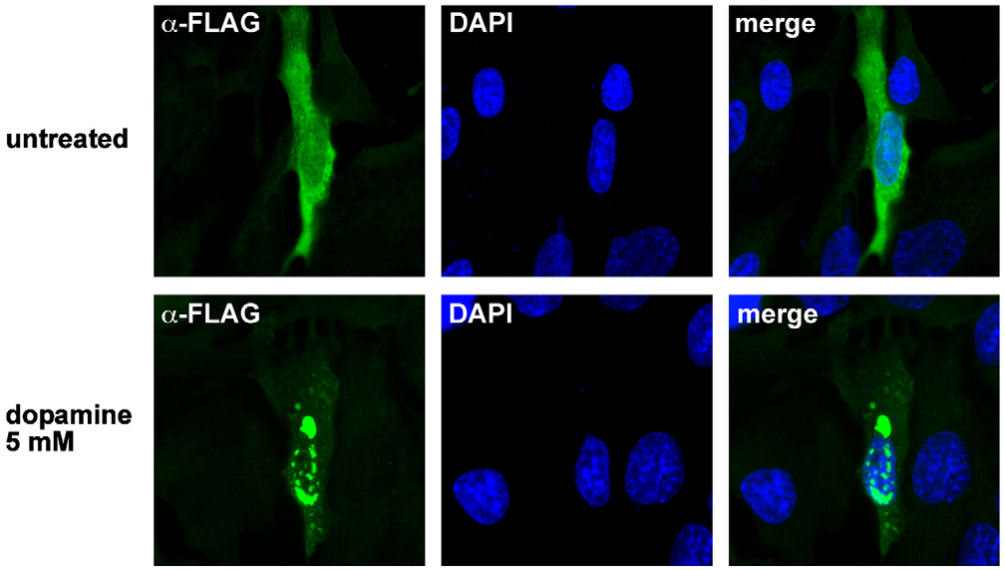
Dopamine induces aggregation of zebrafish parkin. SH-SY5Y cells grown on glass coverslips were transiently transfected with FLAG-tagged zebrafish parkin. 24 h after tansfection, the cells were exposed to dopamine (5 mM, 5 h). The intracellular distribution of zebrafish parkin was analyzed by indirect immunofluorescence of permeabilized cells using an anti-FLAG monoclonal antibody (green). Nuclei were stained by DAPI (blue).

**Figure 4:**
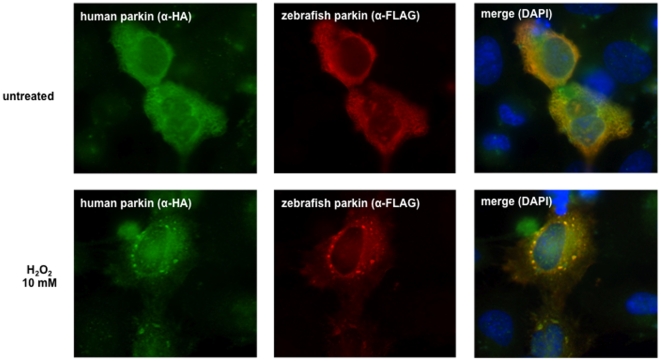
Human and zebrafish parkin co-aggregate. HeLa cells grown on glass coverslips were transiently transfected with FLAG-tagged zebrafish parkin and FLAG-tagged human parkin. 24 h after transfection the cells were exposed to hydrogen peroxide (10 mM) for 30 min. The cells were then fixed and analyzed by indirect immunofluorescence using an anti-HA polyclonal and an anti-FLAG monoclonal antibody. Nuclei were stained by DAPI (blue).

Previous work from our group and other laboratories indicated that parkin is prone to misfolding under high level stress conditions. The propensity to misfold is reflected by a shift of parkin from the detergent-soluble into the detergent-insoluble fraction and the appearance of aggregates [14]. To analyze whether zebrafish parkin shares the propensity to misfold under conditions of oxidative stress, SH-SY5Y cells transiently expressing zebrafish parkin were treated with hydrogen peroxide (10 mMol/L or 20 mMol/L for 30 min). After lysis in detergent buffer (0.1% Triton X-100 in PBS) followed by centrifugation two fractions (supernatant and pellet) were obtained. Whereas zebrafish parkin from untreated cells was primarily found in the detergent-soluble supernatant fraction, hydrogen peroxide induced a shift of zebrafish parkin from the soluble fraction into the detergent-insoluble pellet fraction in a dose-dependent manner (Fig. 2B). In addition to hydrogen peroxide, dopamine has been described to cause a conformational change of human parkin. Detergent-insoluble parkin covalently modified by an oxidation product of dopamine was found in dopamine-treated cultured cells as well as in the brains of PD patients [15]. To analyze the impact of this highly reactive catechol on zebrafish parkin, transiently transfected human SH-SY5Y cells were incubated in the presence of dopamine (0.5, 0.75, 1, or 2 mM for 5 h) and analzed by the detergent solubility assay as described above. Dopamine treatment induced the formation of detergent-insoluble high molecular weight zebrafish parkin aggregates (Fig. 2C). Aggregate formation of zebrafish parkin was also observed by indirect immunofluorescence of cells treated with dopamine (Fig. 3) or hydrogen peroxide (Fig. 4). Notably, human parkin and zebrafish parkin are able to co-aggregate under oxidative stress (Fig. 4).

### Zebrafish parkin is a stress-inducible and stress-protective protein

**Figure 5:**
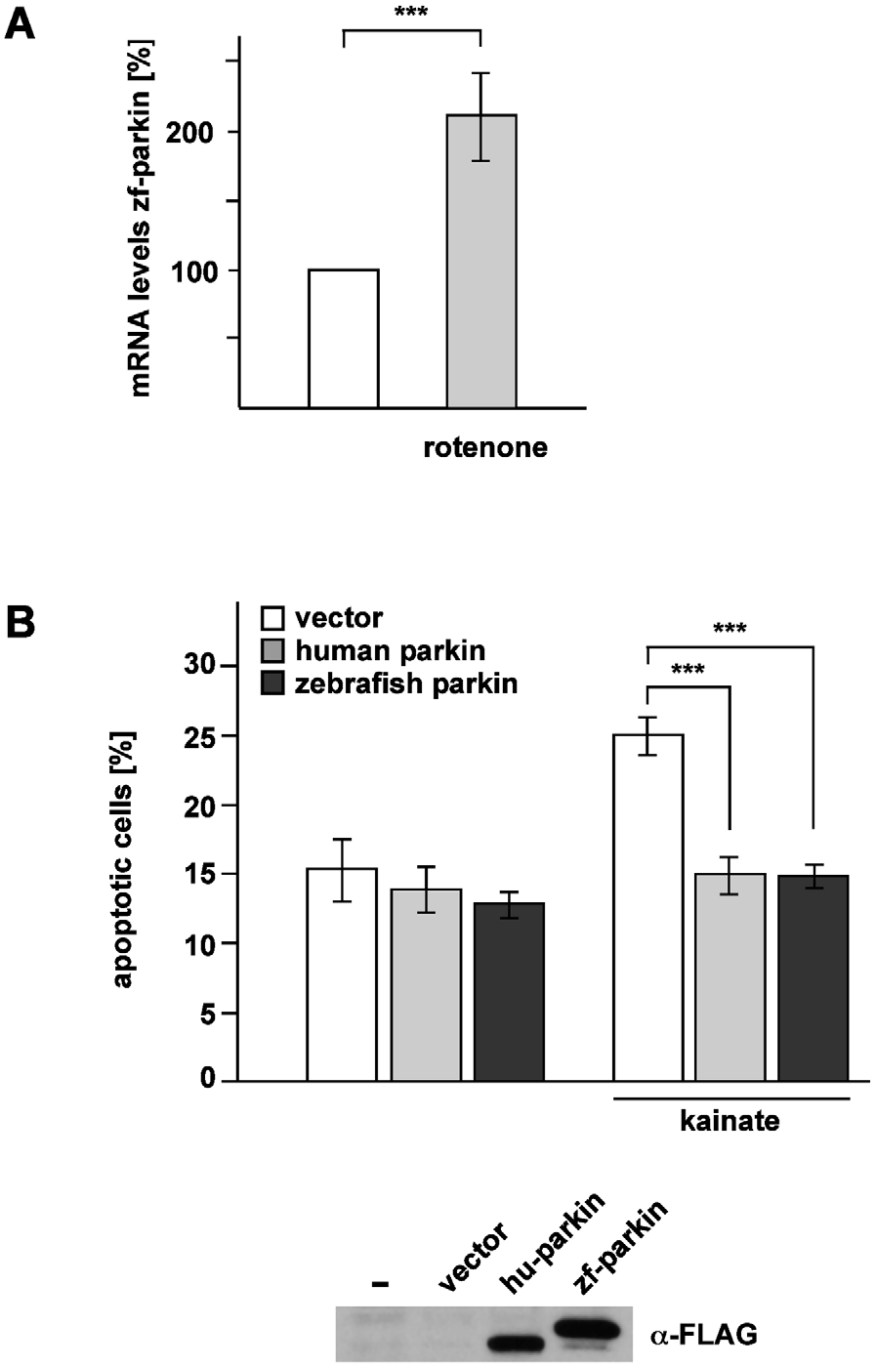
Zebrafish parkin is stress-inducible and stress-protective. (**A**) Zebrafish parkin is transcriptionally up-regulated in response to mitochondrial stress. Pac2 cells (zebrafish fibroblasts) were incubated with rotenone (10 nM) for 1 h and harvested 6 h after treatment. Total cellular RNA was isolated and subjected to semi-quantitative real-time PCR using zebrafish parkin-specific primers. The amount of RNA of each sample was normalized with respect to the endogenous housekeeping control gene β-actin. Shown is the fold increase in the amount of zebrafish parkin-specific mRNA compared with the untreated control based on 5 independent experiments. (**B**) Zebrafish parkin protects cells from excitotoxin-induced cell death. SH-SY5Y cells were co-transfected with EYFP and either vector, FLAG-tagged human parkin (hu-parkin) or FLAG-tagged zebrafish parkin (zf-parkin). 24 h after transfection, the cells were incubated with kainate (1 mM) for 3 h, fixed, permeabilized, and analyzed by indirect immunofluorescence using an antibody against active caspase-3. Shown is the percentage of apoptotic cells among at least 300 transfected cells. The quantification is based on three independent experiments performed in duplicates. As a control for parkin expression, aliquots of the cell lysates were immunoblotted with an anti-FLAG monoclonal antibody (lower panel). ***p<0.001

While parkin is inactivated under high level stress conditions, it has a remarkably wide neuroprotective activity under moderate stress conditions. Parkin has been shown to maintain neuronal integrity under various stressful conditions, including mitochondrial stress, excitotoxicity and ER stress [16], [17], [18]. In support of its crucial role in preventing stress-induced cell death, parkin gene expression is transcriptionally upregulated in response to cellular stress [3], [16], [18], [19], [20], [21], [22], [23], [24], [25], [26]. To analyze whether zebrafish parkin is also a stress-inducible protein, we made use of the zebrafish cell line Pac2, which was treated with rotenone, an inhibitor of complex I of the electron transport chain (10 nMol/L for 1 h). As no anti-parkin antibody is available to detect zebrafish parkin, we quantified endogenous parkin mRNA levels by real-time PCR 6 h after rotenone treatment. In comparison to non-treated control cells we observed a twofold increase in parkin mRNA levels in Pac2 cells exposed to rotenone (Fig. 5A). To test the activity of zebrafish parkin to reduce stress-induced cell death, SH-SY5Y cells transiently expressing human parkin or zebrafish parkin were exposed to the excitotoxin kainate (1 mMol/L for 3 h). Cells undergoing apoptosis were deteced by indirect immunofluorescence using an antibody against active caspase-3. Increased expression of both human and zebrafish parkin protected against the increase in apoptotic cells induced by excitotoxicity (Fig. 5B). Thus, similarly to human parkin, zebrafish parkin is not only a stress-responsive, but also a stress-protective protein.

### Generation of a parkin-deficient zebrafish model

**Figure 6:**
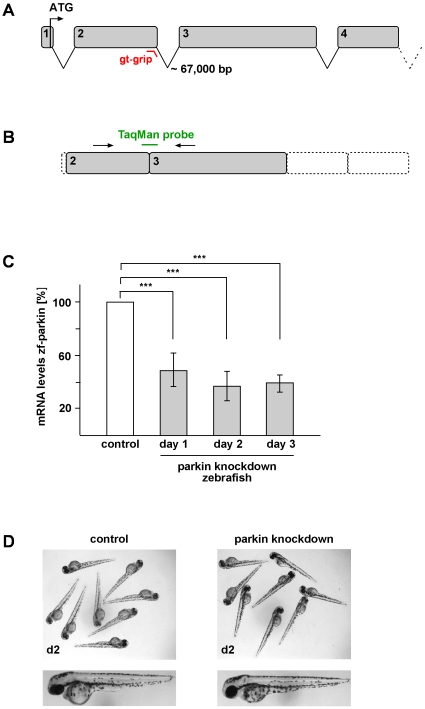
Transient knockdown of parkin in zebrafish does not lead to gross morphological alterations. (**A**) Genomic structure of zebrafish parkin comprising a total of 12 exons. The splice-blocking GT-grip targets the exon-intron junction of exon 2. (**B+C**) Verification of the parkin knockdown efficiency by semi-quantitative real-time PCR. (**B**) The TaqMan™ probe targets the exon 2/exon 3 junction and cannot bind in case of incorrect splicing. (**C**) Parkin knockdown efficiency at day 1, 2 and 3 after injection of the parkin-specific GT-grip in comparison to a control GT-grip. The amount of zebrafish parkin-specific mRNA was normalized to β-actin in parkin GT-grip-injected zebrafish compared to control GT-grip-injected zebrafish. Quantification is based on 4 independent experiments. ***p<0.001 (**D**) Parkin-deficient zebrafish embryos do not show obvious morphological alterations. Two-day-old parkin knockdown zebrafish embryos are shown in comparison to control-injected embryos.

To gain more insight into the *in vivo* function of parkin, a transient knockdown in zebrafish was performed by making use of the antisense technology. Antisense gripNA™ targeting the exon-intron junction of exon 2 (parkin GT-grip) was microinjected into single-cell-stage zebrafish embryos (Fig. 6A). As a control, a mismatch oligonucleotide was injected. GT-grips are described to inhibit pre-mRNA splicing and allow quantification of gene knockdown without the use of antibodies [27]. As a consequence of blocking splice-donor sites three scenarios are possible: 1) the removal of the targeted exon, 2) the use of a cryptic splice donor site, often causing a premature termination codon, or 3) a retention of the targeted intron. To analyze the effects of the parkin GT-grip, total cellular RNA was isolated from zebrafish embryos one day after injection of the parkin GT-grip. Using reverse transcriptase PCR we could rule out the occurance of an alternative splice site. To answer the question whether the parkin GT-grip has an impact on splicing of exon 2 and/or intron 2, we performed semi-quantitative real-time PCR. A TaqMan™ probe was designed to target the exon2/exon3 junction (Fig. 6B). If exon 2 is spliced out and/or intron 2 is not removed the TaqMan™ probe cannot bind correctly and the subsequent decreased signal not only verifies the parkin knockdown, but also enables the quantification of its efficacy. Retention of intron 2 would result in a premature termination codon after codon 65. Loss of exon 2 would cause a frameshift with a premature termination after 4 codons. Due to the fact that exon 1 comprises only 7 base pairs, we were not able to discriminate between these two possibilities. Nevertheless, in either case the parkin protein is severely truncated to either 65 amino acids or 4 amino acids. Analysis of one-day-old zebrafish embryos confirmed splice interference upon parkin GT-grip injection and revealed an overall reduction of parkin mRNA by 53% in zebrafish microinjected with the parkin GT-grip compared to control-injected littermates (Fig. 6C). Notably, the parkin knockdown was stable over at least three days (Fig. 6C). However, we were not able to detect gross morphological or behavioral alterations in parkin knockdown zebrafish (Fig. 6D). Specifically, there were no obvious abnormalities in their swimming behavior and touch response (data not shown).

### Parkin deficiency does not result in a loss of dopaminergic neurons

**Figure 7:**
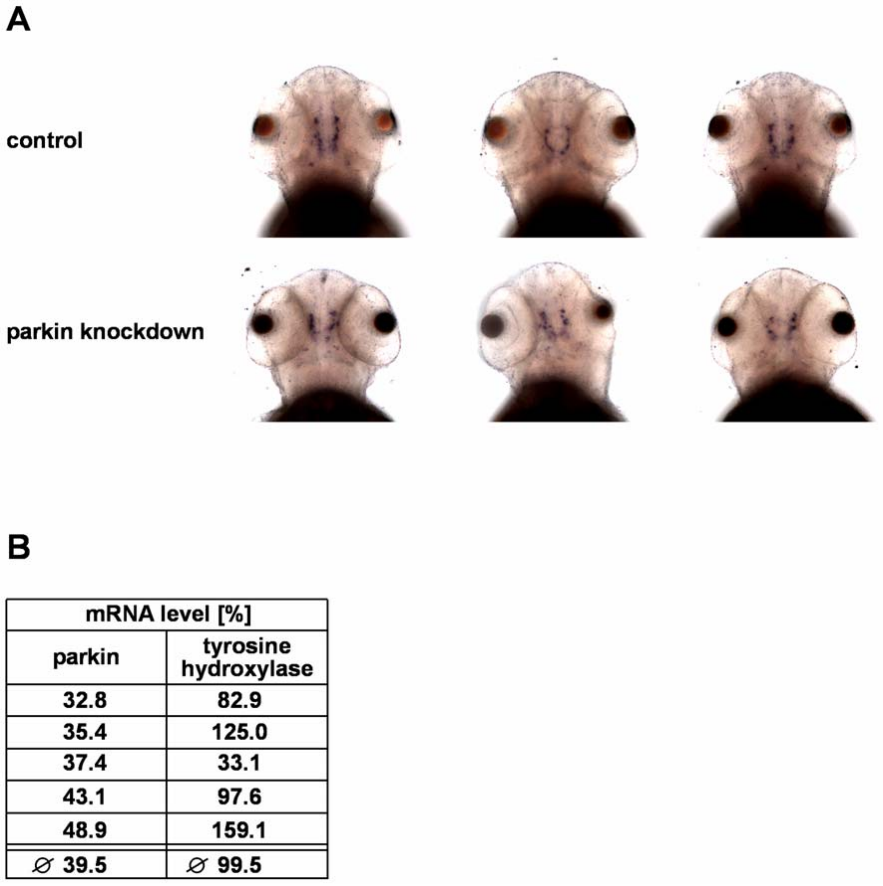
Parkin deficiency has no effect on tyrosine hydroxylase levels in zebrafish. (**A**) *In situ* hybridization using an antisense RNA probe for tyrosine hydroxylase (TH) as a marker for dopaminergic neurons. Staining of parkin knockdown and control-injected zebrafish larvae shows large interindividual variations in both the control and parkin knockdown group. Dorsal views of three-day-old larvae. (**B**) There is no correlation between the efficiency of parkin knockdown and TH mRNA levels. TH mRNA levels were quantified in parkin-knockdown zebrafish using semi-quantitative real-time PCR. The amount of zebrafish TH-specific mRNA was normalized to β-actin in parkin GT-grip-injected zebrafish larvae compared to control-injected embryos at day 3 after injection.The graph shows the results of 5 independent experiments. In each experiment 10–15 zebrafish larvae per group were analyzed.

**Figure 8:**
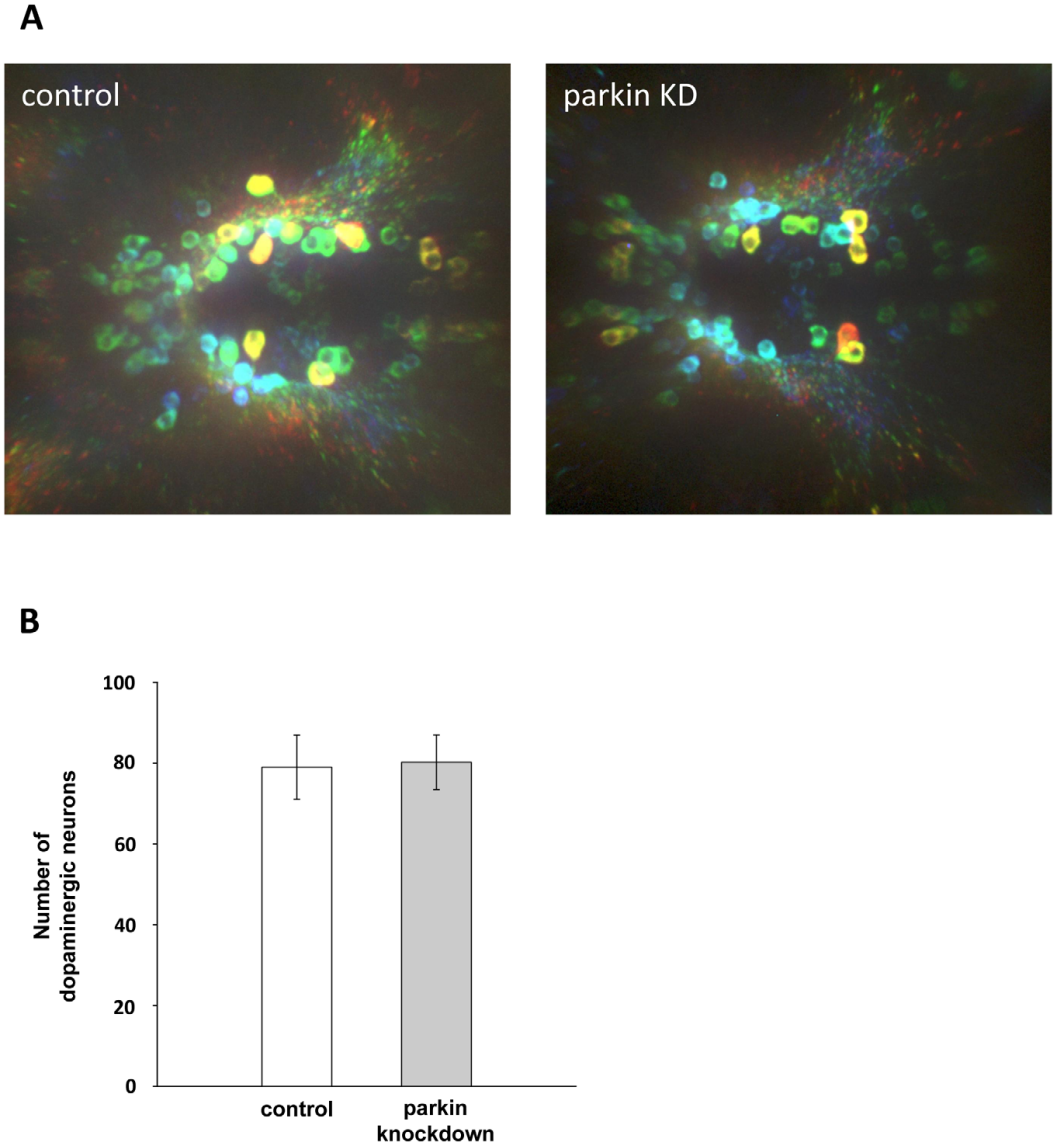
Parkin deficiency does not induce a loss of diencephalic dopaminergic neurons. (**A**) Whole mount immunostaining using an antibody for tyrosine hydroxylase (TH) as a marker for dopaminergic neurons. Staining of control GT-grip-injected and parkin GT-grip-injected zebrafish larvae shows no difference in the number of dopaminergic neurons. The neurons are depth-color-coded to illustrate the vertical position of the individual neurons in the brain and to allow the discrimination of neurons located on top of each other. Red neurons are located most dorsally, followed by yellow, green, blue and violet. Dorsal views of three-day-old larvae, anterior to the left. (**B**) Quantification of TH-positive dopaminergic neurons from 27 control and 27 parkin knockdown zebrafish larvae (parkin knockdown efficiency: 55%). TH-positive neurons were counted in a blinded manner, i.e. the researcher was blind to the knockdown status of the zebrafish larvae.

The teleostean homologue of the mammalian midbrain dopaminergic system is represented by dopaminergic neurons in the diencephalon [28]. Their spatial distribution is established by 72 hours post fertilization [29], [30], [31]. To test for a possible impact of parkin deficiency on dopaminergic neurons in the zebrafish brain, we used *in situ* hybridiziation of an antisense RNA probe for tyrosine hydroxylase (TH), which is the rate-limiting enzyme in the synthesis of dopamine. Parkin knockdown and control-injected zebrafish larvae were subjected to *in situ* hybridization 3 days post fertilization. Microscopic analysis of dopaminergic clusters provided ambiguous results. Interindividual variations in TH staining were not only observed in the parkin-deficient zebrafish cohorts, but also among control-injected larvae (Fig. 7A). We therefore chose an additional approach and determined TH mRNA levels in parkin-deficient zebrafish larvae by semi-quantitative real-time PCR in 5 independent experiments. We observed considerable variations in the TH mRNA levels from experiment to experiment, however, there was no correlation between the parkin knockdown efficiency and TH mRNA levels (Fig. 7B). To extend our analysis on possible effects of parkin deficiency on zebrafish dopaminergic neurons, we performed whole mount immunohistochemistry using an antibody against TH. Parkin knockdown and control-injected zebrafish larvae were stained at day 3 post fertilization. A three dimensional microscopic analysis of the stained dopaminergic clusters by a highly sensitive EMCCD camera revealed that the number of TH-positive neurons was not reduced in parkin-deficient zebrafish larvae, indicating that the partial downregulation of parkin (knockdown efficiency 55%) did not induce a loss of dopaminergic neurons in our zebrafish model. (Fig. 8A, B).

### Downregulation of zebrafish parkin does not affect mitochondrial morphology or membrane potential

**Figure 9:**
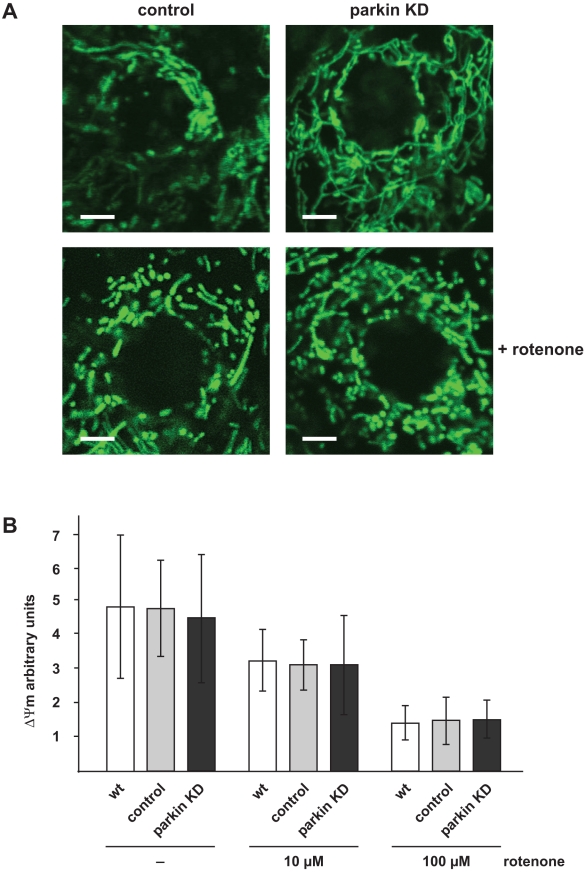
Mitochondrial morphology and membrane potential is not affected in parkin-deficient zebrafish. (**A**) Mitochondrial morphology in not altered in parkin knockdown zebrafish embryos. Live cell imaging of mitochondria in one-day-old parkin-deficient zebrafish and control-injected zebrafish. Mitochondria were visualized by the transient expression of GFP targeted to mitochondria (green). A transient knockdown of parkin in zebrafish did not cause mitochondrial fragmentation in the outer skin of zebrafish embryos (upper images). Mitochondrial fragmentation induced by rotenone (100 µg/l, 6 h) was not aggravated in parkin-deficient zebrafish embryos (lower images). Scale bar 5 µm. (**B**) The membrane potential of total mitochondria isolated from parkin-deficient zebrafish embryos is not altered in comparison to mitochondria isolated from wildtype or control-injected embryos, neither under basal conditions nor after rotenone treatment. Isolated mitochondria from one-day-old wildtype, control-injected and parkin knockdown zebrafish were either left untreated or stressed with different concentrations of rotenone and subsequently incubated with the fluorescent dye JC-1. The mitochondrial membrane potential is represented as the ratio of intra-mitochondrial JC-1 (orange) and cytosolic JC-1 (green). Quantification is based on three independent experiments. KD: knockdown.

Many lines of evidence suggest that mitochondrial dysfunction plays an important role in the pathogenesis of PD [32], [33], [34], [35], [36], [37], [38]. In support of this notion, several PD-associated genes have an impact on mitochondrial integrity. In a previous study we could show that an acute downregulation of parkin in SH-SY5Y cells, primary mouse neurons and *Drosophila* S2 cells affects mitochondrial morphology, membrane potential and function [39]. To test whether a similar phenotype might occur in parkin-deficient zebrafish, we analyzed mitochondria in skin cells of parkin knockdown zebrafish in comparison to control-injected siblings one day post fertilization. Visualization of mitochondria was achieved by the injection of mRNA coding for mitochondrially targeted GFP at the one-cell-stage. Life cell imaging by fluorescence microscopy did not reveal alterations in mitochondrial morphology in epidermal cells of parkin-deficient zebrafish (Fig. 9A, upper images). Next we examined whether mitochondria from parkin knockdown zebrafish are more susceptible to fragmentation in response to rotenone treatment, as observed in cultured fibroblasts from patients with pathogenic homozygous or compound heterozygous parkin mutations [40]. We incubated dechorionated parkin-deficient and control zebrafish embryos with rotenone one day post fertilization (100 µg/l for 6 h). Rotenone-treated zebrafish embryos were characterized by shorter rod-like or spherical mitochondria, but there was no significant difference in mitochondrial fragmentation between control and parkin knockdown zebrafish embryos (Fig. 9A, lower images). Our imaging approach only allowed the assessment of mitochondria within the epidermis, which might be less sensitive to a loss of parkin function. We therefore performed a more comprehensive analysis of mitochondria by isolating total mitochondria from one-day-old parkin knockdown, control and wildtype zebrafish embryos. These mitochondria were analyzed for their membrane potential under basal conditions and in response to rotenone treatment (10 and 100 µM for 50 min) by measuring the uptake of the fluorescent carbocyanine dye JC-1. The ratio of JC-1 in its aggregated intra-mitochondrial form (orange fluorescence) to its monomeric cytoplasmic form (green fluorescence) was used as a quantitative index of mitochondrial function [41], [42]. Rotenone induced a decrease in the mitochondrial membrane potential in a dose-dependent manner, however, we could not detect significant differences between mitochondria isolated from parkin-deficient zebrafish, control-injected or wildtype zebrafish, neither under basal conditions nor in response to rotenone treatment (Fig. 9B).

### Generation and analysis of transgenic zebrafish stably expressing parkin

**Figure 10:**
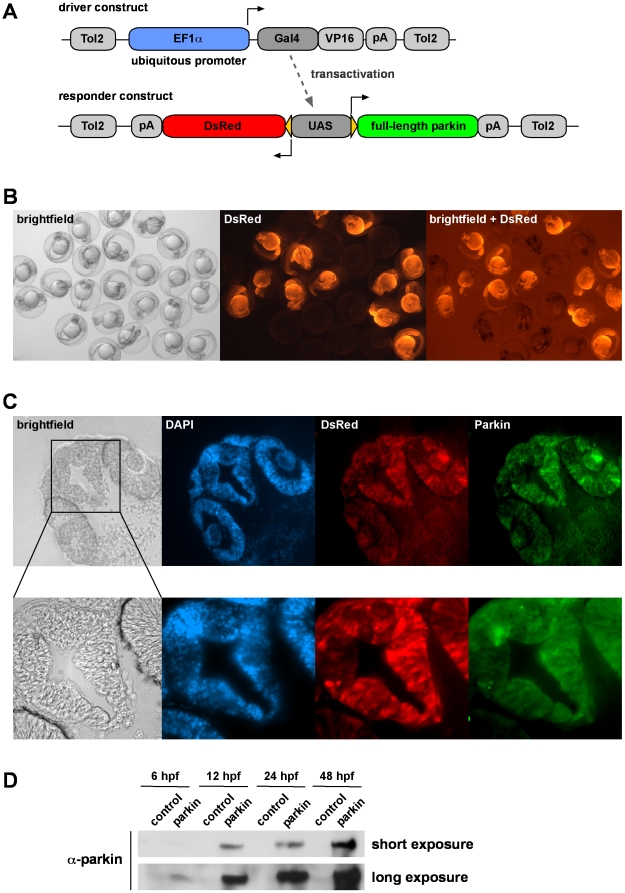
Generation of transgenic parkin zebrafish. (**A**) A Gal4/UAS-based bidirectional expression system consisting of two constructs was used for the generation of transgenic parkin zebrafish. The driver construct contains the ubiquitous promoter EF1α, driving the expression of Gal4/VP16, which binds to the upstream activating sequence (UAS) of the responder construct. Activation of the UAS leads to the expression of both full-length parkin and DsRed. (**B**) DsRed fluorescence in living offspring from transgenic zebrafish outcrossed with wildtype zebrafish. One-day-old transgenic zebrafish characterized by ubiquitous expression of DsRed can be distinguished from their non-transgenic siblings. (**C**) Parkin and DsRed show an overlapping expression pattern in transgenic zebrafish. Cryosections of the head of an one-day-old transgenic parkin zebrafish were analyzed for parkin expression (immunostaining with the anti-parkin mAb PRK8) and DsRed fluorescence. Cell nuclei were stained by DAPI. (**D**) Western blot analysis of transgenic parkin zebrafish and their non-transgenic siblings (control). Embryos were lysed at different time points and parkin present in the lysates was detected by immunoblotting using the anti-parkin mAb PRK8. hpf: hours post fertilization.

To investigate the neuroprotective capacity of parkin in an *in vivo* model, transgenic zebrafish lines stably expressing full-length human parkin were generated. We previously demonstrated that a Gal4/UAS-based bidirectional expression system in zebrafish allows efficient transgenesis, stable inheritance and expression of adequate protein levels combined with a facile identification of transgenic zebrafish due to concomitant expression of the fluorescent protein DsRed [43]. We cloned the Gal4-VP16 coding sequence under control of the ubiquitous promoter EF1α as a driver construct (Fig. 10A). The responder construct comprises the coding sequences of the fluorescent marker DsRed and full-length parkin under control of an upstream activating sequence (UAS). Consequently, the expression of Gal4-VP16 from the driver construct transactivates the UAS of the responder construct, leading to the concomitant transcription of DsRed and parkin. Transgenic zebrafish embryos could be distinguished from their non-transgenic siblings under fluorescent light due to the expression of DsRed (Fig. 10B). Parkin expression was detected by immunohistochemistry in cryosections of one-day-old embryos (Fig. 10C). Parkin was expressed in all tissues analyzed with high expression levels in the brain and eyes. A Western blot analysis revealed that parkin protein was expressed as early as 6 hours post fertilization (Fig. 10D).

### Parkin protects zebrafish from apoptosis induced by proteotoxic stress

**Figure 11:**
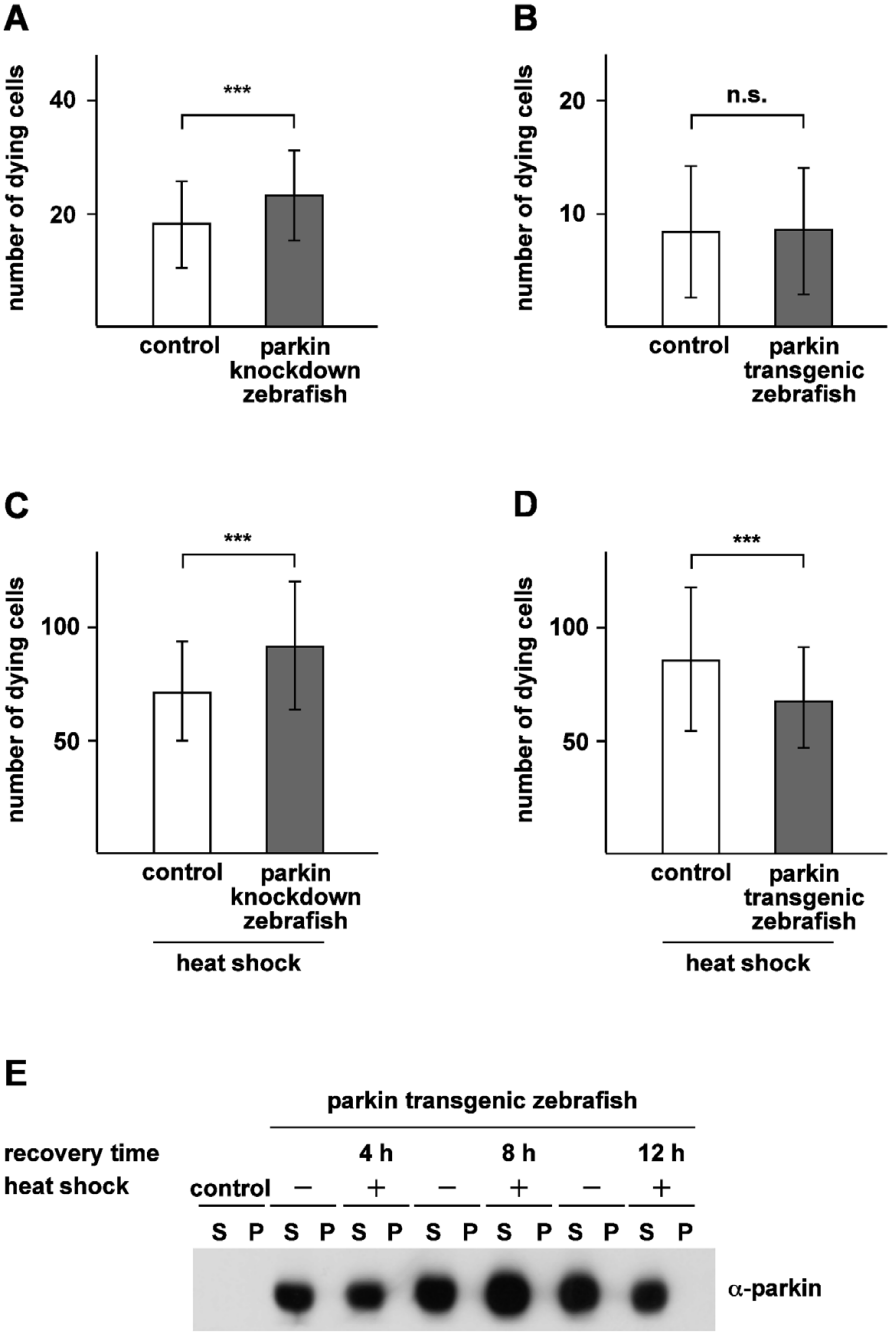
Parkin protects zebrafish from heat shock-induced cell death. (**A**) Basal cell death is slightly increased in parkin-deficient zebrafish. Two-day-old parkin knockdown zebrafish embryos and control-injected embryos were analyzed for dying cells in the spinal cord (neuronal and non-neuronal) by staining with acridine orange. Parkin-deficient zebrafish embryos showed a small but significant increase in basal cell death in comparison to control-injected embryos (23.20±7.96 versus 18.19±7.65 dying cells). (**B**) There is no difference in the rate of cell death between parkin transgenic zebrafish and non-transgenic littermates (8.37±5.82 versus 8.56±5.59 dying cells). (**C+D**) Parkin-deficient zebrafish are more vulnerable to thermal stress, while transgenic parkin zebrafish are more resistant to thermal stress. 48 hours post fertilization zebrafish embryos were exposed to a heat shock (39°C) for 1 h followed by an 8 h recovery period at 29°C. Staining with acridine orange was used to quantify dying cells (neuronal and non-neuronal) in the spinal cord. Cell death in response to thermal stress was increased in parkin knockdown zebrafish compared to control-injected fish (91.47±28,32 versus 71.0±21.96 dying cells) (C) and decreased in transgenic parkin zebrafish versus non-transgenic siblings (85.51±31.81 versus 67.39±22.26 dying cells) (D). Quantification was based on 3 independent experiments. For each experiment at least 50 embryos per group were analyzed. ***p<0.001. (**E**) Thermal stress in zebrafish had no effect on the detergent solubility of parkin. Western blot analysis of two-day-old transgenic parkin zebrafish embryos which were either heat shocked with subsequent recovery periods of 4 h, 8 h and 12 h, respectively, or remained untreated. After lysis of zebrafish embryos in detergent buffer, proteins were fractionated by centrifugation into detergent-soluble (S) and -insoluble (P) fractions, and parkin was analyzed by immunoblotting using the anti-parkin antibody PRK8. Control: non-transgenic zebrafish embryos.

Based on our observation that parkin is able to protect cells from stress-induced cell death in cultured cells, we wondered whether the stress-protective capacity of parkin also applies to the zebrafish model. First, we quantified cell death in the spinal cord in parkin knockdown and transgenic zebrafish under physiological, non-stress conditions. Two-day-old parkin knockdown and parkin transgenic zebrafish embryos were treated with acridine orange in order to detect cell death in living fish. Acridine orange is a vital dye that is specifically taken up in damaged cells and stains nucleic acids in dying cells [44], [45]. Whereas the number of apoptotic cells occuring during normal zebrafish development was not altered in parkin transgenic zebrafish (Fig. 11B), parkin knockdown zebrafish showed a small but significant increase in the number of dying cells compared to control-injected zebrafish embryos (Fig. 11A). The difference in cell death rates between control knockdown zebrafish and control non-transgenic zebrafish is a consequence of the injection procedure, which is a common phenomenon. The slight increase in apoptotic cell death in parkin-deficient zebrafish embryos compared to control-injected embryos obviously causes no morphological or behavioral phenotype, but may indicate an increased general vulnerability of parkin-depleted cells. To follow up this idea, we subjected parkin-deficient and parkin-overexpressing zebrafish to proteotoxic stress. Heat shock is a classical inducer of proteotoxic stress by causing alterations in protein folding, leading to apoptotic cell death when a critical threshold has been exceeded [46]. It has been shown previously that heat shock induces proteotoxic stress also in zebrafish. A temporary elevation of the surrounding temperature by 11°C (from 28°C to 39°C) results in extensive apoptosis in zebrafish embryos throughout the body, particularly in sensitive regions of the brain and spinal cord [47]. Two-day-old parkin knockdown and control-injected zebrafish embryos were exposed to an heat shock by incubating them at 39°C for 1 h followed by a recovery at 28°C for 8 h. Of note, these conditions did not have an effect on the viability, morphology or behavior of zebrafish embryos (data not shown). After a recovery at 28°C for 8 h, the zebrafish embryos were treated with acridine orange, and dying cells were quantified by a microscopic analysis, which revealed a significant increase of cell death in the whole spinal cord of parkin-deficient embryos (Fig. 11C). Next, we performed the same analysis with parkin transgenic zebrafish. Accordingly, two-day-old parkin transgenic zebrafish embryos and their non-transgenic siblings were heat shocked and analyzed as described above. In parkin transgenic zebrafish the number of dying cells upon heat shock was significantly reduced in comparison to non-transgenic siblings (Fig. 11D). Notably, the heat shock conditions applied in our zebrafish model did not cause misfolding of parkin, as determined by a detergent solubility assay (Fig. 11E). Based on the fact that zebrafish live at an ambient temperature of 28°C, an increase to 39°C caused a thermal stress in this organism whithout inducing a conformational change of parkin. In conclusion, parkin-deficient zebrafish are more vulnerable to stress-induced cell death, while parkin transgenic zebrafish are more resistant to cellular stress.

## Discussion

In this study, we made use of the vertebrate *Danio rerio* as an additional vertebrate model organism to study the function of parkin. The zebrafish parkin orthologue is highly homologous to human parkin, particularly within the functional domains and is expressed at all developmental stages up to adulthood. Moreover, zebrafish parkin shows the same biochemical and functional features as human parkin: It is transcriptionally up-regulated in response to cellular stress and protects from stress-induced cell death. We and others have previously shown that human parkin is stress-inducible and stress-protective under moderate stress conditions, while severe oxidative stress induces misfolding and aggreagtion of parkin, leading to its inactivation [14], [15], [48]. In line with these findings, zebrafish parkin adopts a detergent-insoluble conformation and forms cellular aggregates in cells treated with hydrogen peroxide or dopamine.

To study the consequences of parkin deficiency in zebrafish, we used the antisense knockdown technology. The injection of an antisense oligonucleotide targeting the exon-intron junction of exon 2 resulted in a frame shift generating a premature termination codon. The knockdown efficiency determined by semi-quantitative real-time PCR of the normal zebrafish parkin mRNA was between 50 and 60% from day 1 until day 3 after injection, however, we could not detect significant morphological or behavioral alterations in parkin-deficient zebrafish. Specifically, we did not observe a significant loss in diencephalic dopaminergic neurons, neither by *in situ* hybridization with an antisense RNA probe for tyrosine hydroxylase (TH) nor by whole mount immunohistochemistry using an anti-TH antibody. Of note, we observed large interindividual variations in the TH mRNA levels both in the control and parkin-deficient cohorts, but there was clearly no correlation between the efficiency of parkin knockdown and the levels of TH. In line with our findings, degeneration of nigrostriatal dopaminergic neurons could not be found in different parkin knockout mouse strains with a targeted deletion of exon 2, 3 or 7 [6], [8], [9], [49]. Inactivation of parkin in *Drosophila melanogaster* provided controversial results. While Greene et al., Pesah et al. and Saini et al. no did not observe neuronal loss in the dorsomedial, dorsolateral, or anteromedial clusters, Whitworth et al. found a significant reduction of dopaminergic neurons in the protocerebral posterior lateral cluster of parkin-deficient flies [10], [50], [51], [52]. In a different parkin null *Drosophila* model, a reduction of TH immunostaining and shrunken morphology has been observed in dorsomedial dopaminergic neurons [53]. In contrast to our data, a 20% decrease in diencephalic TH-positive cells has recently been reported in another zebrafish model of parkin deficiency [54]. One explanation for this discrepancy could be differences in the knockdown approach. Flinn et al. used a morpholino antisense oligonucleotide targeting exon 9, resulting in a 51 amino acid deletion within the IBR (in between RING) domain. We observed that deletions within the RBR domain, including RING1, IBR and RING2, induce the formation of misfolded, aggregated parkin (unpublished data). Thus, it cannot be excluded that the resulting mutant parkin protein lacking the IBR domain has some toxic potential due to its misfolded state. In support of such a scenario, Flinn et al. observed electron dense material in the t-tubules of fast muscle cells in the trunk somites of their parkin knockdown zebrafish larvae. Another explanation would be differences in the efficiency and duration of the parkin knockdown. As the antisense approach allows only a transient and early downregulation of parkin during the first days of development, we cannot exclude that parkin deficiency at later stages could lead to phenotypic alterations. A knockdown effciency between 50 and 60% models the heterozygous carrier state of parkin mutations rather than the complete loss of function usually found in parkin-associated parkinsonism. It would therefore be interesting to establish stable parkin knockout zebrafish lines by TILLING approaches or by targeted gene mutagenesis using zink finger nucleases [55], [56], [57].

Although our parkin-deficient zebrafish did not display obvious morphological alterations, they were characterized by an increased vulnerability to stress-induced cell death. This observation is in line with the notion that parkin has a neuroprotective activity under cellular stress. In fact, the wide neuroprotective capacity of parkin is the most consistent and reproducible activity of parkin, which correlates best with the the loss-of-function phenotype of pathogenic mutants [32]. Parkin protects cultured cells from cell death induced by excitotoxins, mitochondrial toxins and various other stressors [3], [16], [18], [21], [22], [23], [24], [25], [26]. To better characterize the neuroprotective activity of parkin in an animal model, we generated transgenic zebrafish stably overexpressing parkin. By making use of the Gal4/UAS-based bidirectional expression system we described previously [43], parkin was ubiquitously and stably expressed in transgenic zebrafish as early as 6 hours post fertilization up to adulthood. To establish an appropriate stress model in zebrafish, we tested various toxins, such as mitochondrial toxins (rotenone, CCCP) and excitotoxins (L-glutamic acid, kainate). Unfortunately, the effects of these toxins were difficult to titrate in zebrafish: lower concentrations had no significant effect on cell viability, while at higher concentrations no zebrafish survived. We therefore used thermal stress as a paradigm for proteotoxic stress, which has been decribed to cause apoptosis in zebrafish embryos, particularly in the brain and spinal cord [47]. In response to a transient heat shock, significantly more dying cells were detected in parkin-deficient zebrafish embryos in comparison to littermate controls. Transgenic expression of parkin protected against cell death induced by thermal stress, proving the stress-protective capacity of parkin *in vivo*. It will now be interesting to use the transgenic parkin zebrafish model for a further characterization of the protective capacity of parkin, for example by cross-breeding transgenic parkin zebrafish with other genetic PD zebrafish models.

## Materials and Methods

### DNA constructs

The following clone containing zebrafish parkin was ordered from the RZPD (Deutsches Ressourcenzentrum für Genomforschung): RZPD clone ID: IMAGp998G2015580Q1. The zebrafish parkin coding sequence was subcloned into pCMV Tag 2B vector (Stratagene, La Jolla, USA), allowing the expression of N-terminally FLAG-tagged zebrafish parkin (FLAG-zf-parkin). For the expression of FLAG-tagged human parkin, the coding sequence of human parkin was cloned into the pCMV Tag 2B vector (FLAG-hu-parkin). The HA-ubiquitin construct has been described before [3].

### Antibodies and reagents

The following antibodies were used: anti-ACTIVE caspase-3 pAb (Promega, Mannheim, Germany), cyanine 3 (Cy3)-conjugated anti-rabbit IgG (Dianova, Hamburg, Germany), anti-β-actin mAb (AC-74; Sigma, Taufkirchen, Germany), horseradish peroxidase (HRP)-conjugated anti-mouse and anti-rabbit IgG antibody (Amersham Biosciences, Foster City, CA), anti-HA pAb H6908, anti-FLAG M2 mAb F3165 and anti-FLAG M2 affinity gel, (Sigma), anti-parkin PRK8 mAB (Abcam, Cambridge, USA), anti-GAPDH mAb (AM 4300; Ambion, Austin, USA), anti-HA mAb HRP 3F10 (Roche, Mannheim, Germany), anti-TH pAb (Immunostar, Hudson, USA), AlexaFluor™488 goat anti-mouse IgG, AlexaFluor™488 goat anti-rabbit IgG and AlexaFluor™555 goat anti-mouse IgG (Invitrogen, Karlsruhe, Germany). Rotenone and dopamine, N-ethylmaleamide and JC-1 (5, 5′, 6, 6′-tetrachloro-1, 1′, 3, 3′-tetraethylbenzimidazolcarbocyanine iodide) were purchased from Sigma, kainate from Calbiochem (Darmstadt, Germany), hydrogen peroxide and MG-132 from Merck (Darmstadt, Germany), DAPI (4′–6′-diamidino-2-phenylindole) from Invitrogen, and complete protease inhibitor cocktail from Roche.

### Cell culture and transfection

Human embryonic kidney cells (HEK293T), human neuroblastoma cells (SH-SY5Y) and HeLa cells were cultivated as described previously [14], [58]. The zebrafish embryonic fibroblast cell line Pac2 (Dekens MPS, Current Biology, 2003) were kept in L15 Medium (Leibovitz) from Invitrogen with 15% FCS,100 U/mL penicillin and 100 mg/mL streptomycin. Cells were transfected by a liposome-mediated method using Lipofectamine Plus Reagent (Invitrogen) according to the manufacturer's instructions.

### Western Blotting

Proteins were size-fractionated by SDS-PAGE (10% polyacrylamide) and transferred to nitrocellulose (Protran BA 85, Schleicher & Schuell, Dassel, Germany) by electroblotting. The nitrocellulose membranes were blocked with 5% nonfat dry milk in PBST (PBS containing 0.1% Tween 20) for 30 min at room temperature and subsequently incubated with the primary antibody diluted in PBST for 16 h at 4°C. After extensive washing with PBST, the membranes were incubated with horseradish peroxidase-conjugated secondary antibody for 40 min at room temperature. Following washing with PBST, the antigen signal was detected with the enhanced chemiluminescence detection system (Amersham Biosciences) as specified by the manufacturer.

### Ubiquitylation assay

HEK293T were transiently co-transfected with HA-tagged ubiquitin and either pCMV Tag2B vector, pCMV Tag2B human parkin (FLAG-hu-parkin) or pCMV Tag2B zebrafish parkin (FLAG-zf-parkin). The cells were incubated with 5 µM MG-132 over night. One day after transfection, protein extracts were prepared in denaturing lysis buffer (50 mM Tris/HCl, pH 7.4, 5 mM EDTA, 1% SDS, 15 U/ml DNase I, protease inhibitor cocktail and 25 mM N-ethylmaleamide) and boiled for 5 minutes. Protein extracts were diluted 1∶10 with non-denaturing lysis buffer (50 mM Tris/HCL pH 7.4, 300 mM NaCl, 5 mM EDTA, 1% Triton X-100, and protease inhibitor cocktail). After incubation on ice followed by centrifugation the supernatant was pre-cleared by shaking with agarose beads at 4°C for 1 h. Aliquots from the supernatant were taken as an input control, the rest was incubated with anti-FLAG M2 agarose beads overnight in order to pull down FLAG-zf-parkin and FLAG-hu-parkin. After washing three times with non-denaturing lysis buffer and TBS the proteins present in the immunoprecipitates were released from the FLAG-M2 agarose beads by addition of Laemmli sample buffer containing 1% SDS and heating at 95°C for 5 min. Proteins present in the immunoprecipitates were analyzed by Western blotting using the anti-HA mAb HRP 3F10.

### Caspase-3 Assay

SH-SY5Y cells were grown on glass coverslips and co-transfected with EYFP and either vector, human FLAG-parkin or zebrafish FLAG-parkin. 24 h after co-transfection, cells were treated with kainic acid (1 mM) for 3 h. Subsequently, cells were fixed with 4% paraformaldehyde for 30 min, permeabilized with 0.2% Triton X-100 for 10 min at room temperature, and blocked with 5% BSA, 0.1% Tween 20 in PBS for 1 h at room temperature. Fixed cells were incubated with anti-ACTIVE caspase-3 antibody overnight at 4°C, followed by an incubation with Cy3-conjugated secondary antibody for 1 h at room temperature. After washing with PBS cells were mounted onto glass slides and examined by fluorescence microscopy using a Zeiss (Jena, Germany) Axioscope2 plus microscope. To detect cells undergoing apoptosis, the number of activated caspase-3 positive cells of a minimum of 300 transfected cells was determined. Quantifications were based on duplicates of at least three independent experiments.

### Indirect immunofluorescence experiments

SH-SY5Y or HeLa cells were grown on glass coverslips. 24 h after transfection with FLAG-zf-parkin cells were incubated in 5 mM rotenone for 5 h. Afterwards the cells were fixed in 50% aceton/50% methanol for 1 min at room temperature and blocked with 1% BSA in TBS for 2 h at room temperature. Fixed cells were incubated with anti-FLAG M2 mAb and/or anti-HA pAb overnight at 4°C, followed by an incubation with AlexaFluor™ goat anti-mouse IgG and/or AlexaFluor™ goat anti-rabbit IgG for 2 h at room temperature. After washing with TBS, cells were mounted onto glass slides and examined by fluorescence microscopy using a Zeis Axioscope2 plus microscope. Nuclei were stained with 4′, 6′-diamidino-2-phenylindole (DAPI).

### Semi-quantitative real-time PCR

Pac2 cells were incubated with 10 nM rotenone for 1 h and harvested 6 h after drug treatment. Total cellular RNA was isolated and samples were treated with DNase I according to the instructions of the manufacturer (RNeasy mini kit; Qiagen, Hilden, Germany). Zebrafish embryos and larvae were frozen in liquid nitrogen at 1, 2 or 3 days post fertilization. 10–20 embryos or larvae, respectively, were lysed in 1 ml lysis buffer containing 10 µl β-mercaptoethanol and homogenized by passing through a needle. After high speed centrifugation the supernatant was mixed 1∶1 with 70% ethanol. The following centrifugation, DNase digestion and washing steps on the RNeasy mini column were performed according to the manufacturer's instruction. cDNA was synthesized using High-Capacity cDNA Reverse Transcription Kit (Applied Biosystems). Each semi-quantitative real-time PCR sample (20 µl) included 9 µl of cDNA solution, 10 µl of 2x TaqMan™ Universal PCR Master Mix, and 1 µl working stock of TaqMan Gene Expression Assay containing zf-Parkin-E23F (forward): ACGAGAGCTCTGCAATGAATCC, zf-Parkin-E23R (reverse): ACATGAACCGTGCTCTGCTC, zf-Parkin-E23M (TaqMan™ probe): ATCACAGCCCTGAAGTGT; zf-b-actin1-E45F (forward): GCTCTCTCCAGCCTTCCTT, zf-b-actin1-E45R (reverse): TCGCACTTCATGATGGAGTTGA, zf-b-actin1-M45 (TaqMan probe): CTGGGTATGGAATCTT; zf-tyrosine hydroxylase-E56F: GGAGAACCAATTCCTAGAGTGGACTA, zf-tyrosine hydroxylase-E56R (reverse): GATCTCGGAGGGTGGAGTAGA, zf-tyrosine hydroxylase-E56M (TaqMan™ probe): CCTCCCGCCATGTTC. Quantification was performed with 7500 Fast Real Time System (Applied Biosystems) using triplicates for each primer set and each RNA sample. RNA expression was normalized with respect to zf-β-actin. Relative expression was calculated for each gene using the DDCt method.

### Stress treatment and detergent solubility assay

24 h after transfection SH-SY5Y cells were incubated in PBS containing 10 or 20 mM H_2_O_2_ for 30 min or 0.5, 0.75, 1, 2, or 5 mM dopamine for 5 h. Cells were washed twice with cold PBS, scraped off the plate, pelleted by centrifugation, and lysed in cold detergent buffer (0.1% Triton X-100 in PBS). The lysate was centrifuged at 15,000× g for 20 min at 4°C. The supernatant fraction was then separated from the pellet fraction. After adding Laemmli sample buffer to both fractions the samples were boiled for 10 min. To compare the relative distribution of zebrafish parkin, equal amounts of the detergent-soluble and -insoluble fractions obtained from untreated or stressed cells were analyzed by immunoblotting. To analyze the detergen solubility of parkin in heat shocked zebrafish, transgenic parkin zebrafish and their non-transgenic siblings were heat shocked 48 h post as described below. The zebrafish were allowed to recover at 28°C for 4 h, 8 h and 12 h. Cohorts of 20 embryos were lysed in detergent buffer (0.2% Triton X-100 in PBS containing complete protease inhibitor cocktail [Roche, Mannheim, Germany; diluted 1∶100] and passed thirty times through a 27-gauge needle. After centrifugation at 15,000× g at 4°C for 20 min the supernatant was separated from the pellet and further processed as described above.

### Zebrafish Maintenance and Breeding

Zebrafish were maintained, mated, and raised as described [59]. Embryos were kept in embryo medium (5 mM NaCl, 0.17 mM KCl, 0.33 mM CaCl_2_, 0.33 mM MgSO_4_) at 28°C and staged as previously shown [60]. The wildtype line AB, TLF and albino were used for all experiment (www.zfin.org).

### Antisense gripNAs™-mediated knockdown of parkin

Sequence-specific antisense oligonucleotides of 18 nucleotides in length (gripNAs™) were obtained from Activ motif (Carlsbad, USA). For parkin we used a grip hybridizing to a splice-donor-site of an exon-intron junction, resulting in a nonsense splicing product (GT-grip). As control a mismatch GT-grip was designed. Zf-Parkin-GT2-grip: TTACCTGAAGTGTGGATT. Zebrafish embryos were microinjected with a Femto Jet Microinjector (Eppendorf, Hamburg, Germany) at the one-cell-stage with 13 ng gripNA™. After injection the zebrafish embryos were maintained in embryo medium at 28°C.

### Transgenic parkin zebrafish

To generate transgenic zebrafish expressing DsRed and human parkin, the full-length human parkin cDNAwas amplified from a pCS2+ vector (Rupp RA, Genes Dev., 1994) by PCR using primers hP-XbaI-F: 5′-GCTCTAGAGCCACCATGATAGTGTTTGTCAG-3′ and d179-XbaI-R: 5′-TGCTCTAGACTACACGTCGAACCAGTGGTCCC-3′. The amplicon was cloned into the PCR8/GW/TOPO/Entry vector (Invitrogen). The insert of the entry vector was subcloned into the destination responder vector by LR-recombination using the gateway recombination system in order to obtain a protein expression vector [43]. EF1α-Gal4-VP16 was amplified by PCR from pBEF1α-Gal4-VP16 (kindly provided by R. W. Köster) using the primers MI3-FP: TGTAAAACGACGGCCAGT and MI3-RP: CAGGAAACAGCTATGACC and TOPO-cloned into pCR8/GW-ENTR/TOPO. Then, EF1α-Gal4-VP16 was transfered into pT2d-DEST-GwRI-R2 by LR-recombination. The resulting plasmid pT2d-EXP- EF1α-Gal4-VP16.pA was used to generate transgenic zebrafish. All the further experimental steps including the transformation of *E.coli* DH5alpha were done according to the instructions of the Gateway Clonase LR II enzyme kit (Invitrogen). Plasmid DNA was prepared and purified for injection into zebrafish embryos using the Geneclean II Kit (Qbiogene, Carlsbad, USA). In order to obtain transposase mRNA, plasmid DNA was linearized and purified by phenol/chloroform extraction. mRNA was synthesized using the Ambion mMESSAGE mMachine Kit (Applied Biosystems) according to the manufacturer's protocol. Shortly before the injection, purified DNA constructs were diluted with DEPC (diethylenepyrocarbonate)-treated ultrapure water to an approximate concentration of 15 ng/µl for the driver construct and 40 ng/µl for the responder construct. In parallel, transposase mRNA was diluted to 20 ng/µl in DEPC water. DNA and mRNA dilutions were mixed in a 1∶1 ratio and phenol red was added to a final concentration of 20% (v/v). One-cell-stage fertilized zebrafish eggs were injected with about 1 nl of the injection mixture. Expression of transposase after mRNA injection catalyzes the integration of both the driver and responder construct into the zebrafish genome via the Tol2 sites [61]. Injected embryos were screened for DsRed expression between 24 to 48 hours after fertilization using fluorescence microscopy. Zebrafish embryos positive for DsRed expression and with a normal appearance were sorted and further raised to adulthood. Adult founder zebrafish were identified by outcrossing them to AB/Albino/TLF fish (generously provided by L. Bally-Cuif) and screening the F1 generation for DsRed-expressing embryos. These embryos were raised to establish stable transgenic lines.

### Cryosection and immunostainings of zebrafish embryos

For immunostaining of parkin, zebrafish embryos were fixed in 4% paraformaldehyde overnight. After short washing in PBS single fish were embedded in Tissue-Tek OCT compound (Sakura Finetek Europe, Zoeterwoude, The Netherlands) and frozen on dry ice. 14 µm thin sections were cut on a Microm HM 560 cryotome (Thermo Scientific, Walldorf, Germany) at −22°C and collected on microscopic slides. Embedding medium was removed by washing the slides with PBS containing 0.5% Tween-20. The slides were blocked in 10% NCST in PBST (10% newborn calf serum, 0.1% Tween and 1% DMSO in PBS) for two hours, then the anti-parkin mAb PRK8 was diluted 1∶200 in 2% NCST and incubated overnight at 4°C. After incubation with anti-mouse Alexa-488 antibody for three hours at room temperature the slices were washed in PBST and PBS. Mowiol mounting medium (Calbiochem) was homogenously distributed and the slices were covered with coverslips. The sections were analyzed using a Zeiss Axioscope2 plus microscope. For immunostaining of TH, 3-day-old zebrafish were fixed overnight in 4%PFA, transfered to methanol, rehydrated in PBST, transfered to distilled water, incubated in icecold aceton for 7 min at −20°C, blocked with NCST and incubated with the anti-TH antibody diluted in NCST (1∶200) overnight. The first antibody was detected with an anti-mouse-Alexa-488 antibody overnight. Larvae were transfered to 75% glycerol in distilled water, mounted in 50% glycerol/1% agarose in distilled water and analyzed by microscopy.

### Imaging of dopaminergic neurons in zebrafish larvae

Three-day-old larvae stained with an anti-TH antibody (Immunostar, Hudson, USA) were analyzed using a Zeiss Cell Observer Spinning Disc microscope. Images were taken using a Zeiss LD LCI Plan-Apochromat 25x/0.80 objective with glycerol immersion and a Photometrics Evolve 512 EMCCD camera. In parallel, larvae from the same injected clutch were subjected to quantitative real time PCR in order to determine the parkin knockdown efficiency. Dopaminergic neurons of populations 1 to 6 in the posterior tuberculum (according to terminology developed by Rink and Wullimann [62] were counted in parkin GT-grip-injected embryos and mismatch GT-grip-injected embryos in a blinded manner, i.e. the researcher was blind to the knockdown status. These dopaminergic neurons include the populations ascending to the striatum (populations 1, 2 and 4). As these neurons lie in different layers of the brain, a Z-stack of the brain was taken, which contained all neurons of these populations. A 3D image was projected and depth-color coded using the Zeiss Axiovision software. The depth coloring allows to discriminate neurons lying perpendicular on top of each other.

### Heat shock

Cohorts of 20 zebrafish embryos were put into a volume of 1 ml embryo medium in 2 ml Eppendorf cups. Cups with open lids were placed in a heating block at 39°C for 1 h. Stress treatment was administered 48 h post fertilization and embryos were allowed to recover at 28°C for 4, 8 and 12 h, respectively.

### *In situ* hybridization

5 µg of pB-SK zTH (kindly provided by W. Driever) were linearized with XhoI at 37°C overnight and cleaned up using phenol/chloroform. 1 µg of purified DNA was subjected to Digoxigenin-RNA synthesis following the manufacturer's instruction (DIG RNA Labeling Kit, Roche) and T3 RNA polymerase (Fermentas, St. Leon-Rot, Germany). After precipitation the pellet was resuspended in DEPC-treated ultrapure water, 20× SSC (standard saline citrate) and formamide. Zebrafish larvae were fixed in 4% paraformaldehyde (PFA) overnight at 4°C 3 days post fertilization. They were washed twice in PBST for 5 min at room temperature. After digestion with proteinase K in PBST (10 µg/ml) (Roche) for 20 min larvae were postfixed in 4% PFA for 20 min at room temperature and then washed twice in PBST for 5 min at room temperature. Larvae were pre-hybridized in hybridization buffer (50% formamide, 5× SSC, 0.1% Tween, 50 µg/ml heparin, 5 mg/ml torula yeast RNA) at 65°C for 2 h. Hybridization was carried out overnight at 65°C in hybridization buffer containing tyrosine hydroxylase single-stranded RNA probe labeled with digoxigenin-UTP (Roche). All subsequent washing steps were carried out at 65°C. Washes were performed as follows: 2×30 min in 50% formamide, 2 x SSCT (standard saline critrate +0.1% Tween); 1×15 min in 2× SSCT; 2×30 min in 0.2× SSCT; 1×10 min in 66% 0.2× SSCT, 33% PBST; 1×10 min in 33% SSCT, 66% PBST, 1×10 min in PBST. Larvae were blocked for 1 h at room temperature in blocking buffer (10% fetal calf serum, 1% DMSO, 0.1% Tween 20 in PBS) and subsequently shaken overnight at 4°C in pre-absorbed Fab-alkaline phosphatase (Roche) in blocking buffer (1∶4000). Larvae were washed three times in staining buffer for 10 min at room temperature. To reduce endogenous phosphatase activity, 0.25 mg/ml levamisole (Sigma) was added to the staining buffer (100 mM Tris pH 9.5, 50 mM MgCl_2_, 100 mM NaCl, 0.1% Tween 20). Staining was carried out with nitroblue tetrazolium chloride (NBT) (Sigma) and 5-bromo 4-chloro3indolyl phosphate (BCIP) (Roche) in staining buffer for 30 min. Pictures were taken using a Zeiss compound microscope with a Zeiss Plan-Apochromat 10×/0.45 lens. Z-stack images were generated by scanning through the brain at an optimal image slice distance and combined in a maximum projection.

### Acridine orange staining

2 days post fertilization zebrafish embryos were dechorionated, anesthetized with tricaine (Sigma) and incubated alive in 9 µg/ml acridine orange (Sigma) in embryo medium for 30 min at room temperature. Stained embryos were analyzed after 3 wash steps of 5 min with embryo medium. For imaging, zebrafish embryos were embedded in 1.5% low melting agarose and overlayed with embryo medium containing tricaine. The neurons with fluorescent green nuclei undergoing cell death were counted in the whole spinal cord using a Zeiss compound microscope with a Zeiss Plan-Apochromat 10×/0.45 lens. Counting of 30 to 70 embryo of each group was performed in a blinded manner.

### Live imaging of mitochondria in zebrafish

Embryos were microinjected at the one-cell-stage with gripNA™, mRNA coding for GFP containing a mitochondrial targeting sequence (mito-GFP, which was synthesized from pCS2+mito-GFP after subcloning from pYX223mtGFP [63]). For some experiments we additionally microinjected the mRNA of the histone protein H2B containing the coding sequence of the monomeric red fluorescent protein, in order to stain cell nuclei. One day post fertilization dechorionated embryos were incubated with rotenone (100 µg/L for 6 h). For imaging, anesthetized zebrafish embryos were embedded in 1.5% low melting agarose and overlayed with embryo medium containing tricaine. Pictures were taken using a Zeiss LSM Meta Confocal microscope.

### Isolation of mitochondria from zebrafish

Crude mitochondrial and cytoplasmic fractions were isolated using a Mitoiso 1 isolation kit (Sigma). All steps were performed at 4°C with icecold buffers. 24 hours post fertilization 30 embryos were dechorionated, anesthetized and homogenated in the supplied extraction buffer by sonication. Homogenates were spun at 600× g for 5 min at 4°C, and then the supernatants were spun at 11,000× g for 10 min at 4°C. The resulting pellet was resupended in extraction buffer and the centrifugation steps were repeated. The final pellets representing the mitochondrial fraction were resuspended in the supplied storage buffer.

### Measurement of the mitochondrial membrane potential

The mitochondria fractions were either left untreated or were incubated with the mitochondrial complex I inhibitor rotenone (10 µM or 100 µM) for 10 min at room temperature. The integrity of the mitochondrial membrane potential was assessed by measuring the uptake of fluorescent dye 5, 5′, 6, 6′-tetrachloro-1, 1′, 3, 3′-tetraethylbenzimidazolcarbocyanine iodide (JC-1). The mitochondria fractions were incubated with the supplied JC-1 Assay buffer (Sigma) and JC-1 for 10 min at room temperature following the manufacturer's instruction. The fluorescence was detected using a Fluoroskan Ascent plate reader (Sigma): excitation 485 nm/emission 538 nm for green fluorescence of monomeric cytosolic JC-1 and excitation 530 nm/emission 590 nm for orange fluorescence of intramitochondrial JC-1 aggregates. The ratio of 590/538 nm (orange/green) was considered as an indicator of mitochondrial membrane potential. Quantifications were based on triplicates of three independent experiments.

### Ethics Statement

All zebrafish husbandry and experimental procedures were performed in accordance with the German animal protection standards and were approved by the Government of Upper Bavaria (Regierung von Oberbayern, Munich, Germany).

### Statistical analysis

Mean values and standard deviations (SD) were calculated with Microsoft Excel. Statistical analysis was performed using 2-tailed Student's t test, *p<0.05, **p<0.01, ***p<0.001. Data are presented as mean ± SD.
